# An *in vitro* model for the cultivation of polymicrobial biofilms under continuous-flow conditions

**DOI:** 10.12688/f1000research.55140.1

**Published:** 2021-08-13

**Authors:** Thomas James O'Brien, Marwa Mohsen Hassan, Freya Harrison, Martin Welch

**Affiliations:** 1 Department of Biochemistry, University of Cambridge, Cambridge, CB2 1QR, UK; 2 School of Life Sciences, University of Warwick, Coventry, CV4 7AL, UK

**Keywords:** Cystic fibrosis (CF), polymicrobial, biofilms, in vitro models, 3Rs

## Abstract

The airways of people with cystic fibrosis (CF) are often chronically colonised with a diverse array of bacterial and fungal species. However, little is known about the relative partitioning of species between the planktonic and biofilm modes of growth in the airways. Existing *in vivo* and *in vitro* models of CF airway infection are ill-suited for the long-term recapitulation of mixed microbial communities. Here we describe a simple, *in vitro *continuous-flow model for the cultivation of polymicrobial biofilms and planktonic cultures on different substrata. Our data provide evidence for inter-species antagonism and synergism in biofilm ecology. We further show that the type of substratum on which the biofilms grow has a profound influence on their species composition. This happens without any major alteration in the composition of the surrounding steady-state planktonic community. Our experimentally-tractable model enables the systematic study of planktonic and biofilm communities under conditions that are nutritionally reminiscent of the CF airway microenvironment, something not possible using any existing *in vivo* models of CF airway infection.

Research highlights **Scientific benefits**

- Allows for direct comparison of biofilm and planktonic microbial lifestyles.
- Allows for longitudinal and real-time analysis of interspecies interactions among polymicrobial biofilms.

**3Rs benefits**

- Reduces the need for vertebrate infection models when studying microbe-microbe interactions in the context of cystic fibrosis.

**Practical benefits**

- Inexpensive and simple to operate.
- Chemically defined culture conditions, allows for the reproducible study of subtle interspecies interactions.
- Experimentally tuneable, allows for different species combinations or disease states to be studied.

**Current applications**

- Studying changes in ecology of polymicrobial biofilm formation during co-culture on different solid substrata.

**Potential applications**

- Studying changes in gene expression and behaviour of polymicrobial biofilms comprising of different species combinations.
- Studying response of polymicrobial biofilms and planktonic communities against treatment with antimicrobials for the development and validation of polymicrobial biofilm dispersal/treatment regimens.

## Introduction

Cystic fibrosis (CF) is a life-limiting genetic disorder estimated to affect 70,000 people worldwide ( Cystic Fibrosis Foundation). Although a systemic multi-organ disease, the most striking manifestation of CF is chronic obstruction of the airways *via* an overproduction of viscous, nutrient-rich airway secretions. These secretions block the airways and predispose people with CF to life-long microbial infection. These infections are frequently polymicrobial and comprise both bacterial and fungal species ( Ahmed et al., 2019; Boutin et al., 2015; Carmody et al., 2015; Hogan et al., 2016; Jorth et al., 2019; Mahboubi et al., 2016; Rogers et al., 2010; Sibley and Surette, 2011; Zhao et al., 2012). CF airway infections contribute towards a decline in pulmonary function and it is estimated that ~90% of persons with CF succumb to respiratory failure as a direct result of microbial infection ( Chmiel and Davis, 2003; Elborn, 2016; Lubamba *et al*., 2012; Lyczak *et al*., 2002; Rajan and Saiman, 2002; Sibley *et al*., 2006).

Recent years have seen increasing recognition that inter-species interactions between the airway microbiota may play a role in modulating the behaviour, virulence, and even the response to therapeutic intervention ( Antonic *et al*., 2013; Armbruster *et al*., 2016; Baldan *et al*., 2014; Barnabie and Whiteley, 2015; Beaume *et al*., 2015; Briaud *et al*., 2019; Dalton *et al*., 2011; Diggle *et al*., 2007; Elias and Banin, 2012; Hotterbeekx *et al*., 2017; Hibbing *et al*., 2010; Korgaonkar *et al*., 2013; Mastropaolo *et al*., 2005; O'Brien and Fothergill, 2017; Peters *et al*., 2012; Weimer *et al*., 2010). Moreover, co-culturing bacterial species *in vitro* and *in vivo* has been shown to lead to significant alterations in the expression of core essential genes ( Ibberson *et al*., 2017; Ibberson and Whiteley, 2020). One particularly important unaddressed question relates to the impact of co-habiting species on the biology of *Pseudomonas aeruginosa* (PA). PA is a common inhabitant of the CF airways, and a model organism for the study of biofilms. However, the paucity of experimentally tractable *in vivo* or *in vitro* models of CF infection has severely hampered the in-depth and longitudinal study of such polymicrobial communities ( O'Brien and Welch, 2019b).

It has been estimated that at least 60% of bacterial infections in the western world involve the formation of biofilms ( Fux *et al*., 2005) and CF is no exception. One crucial feature of microbial biofilms is their increased ability to bypass effective immune clearance and resist antimicrobial action. Indeed, some bacterial biofilms are up to 1000 × more resistant to antimicrobial intervention compared with their planktonic cell counterparts ( Parsek, 2003). The formation of biofilm-like aggregates in the CF airways is often cited as a reason why therapeutic strategies aimed at eradicating keystone CF pathogens such as PA fail ( Bjarnsholt *et al*., 2009; Döring *et al*., 2011; Elias and Banin, 2012; Folkesson *et al*., 2012; Leekha *et al*., 2011; Lopes *et al*., 2012; Lopes *et al*., 2014; Mowat *et al*., 2011). However, our understanding of how inter-species interactions alter biofilm physiology remains limited. This is important because agonistic and antagonistic interactions between species almost certainly confer a significant selection pressure, thereby driving adaptive divergence in members of the polymicrobial community ( Markussen *et al*., 2014; Schick and Kassen, 2018; Winstanley *et al*., 2016).

There are three key vertebrate models of CF available to researchers: the CF mouse, the CF ferret and the CF pig (reviewed recently by O'Brien and Welch, 2019b). Although the porcine airways share a remarkable degree of genetic and structural homology with human airways ( Judge *et al*., 2014; Rogers *et al*., 2008), the cost and technical/ethical complexity of using porcine models ensures they are rarely used in research. CF ferrets are also rarely used, since they develop severe airway infections soon after birth and subsequently succumb to respiratory failure ( Hoffman and Hajjar, 2018; Sun *et al.*, 2010), although this situation may change as *in utero* administration of cystic fibrosis transmembrane conductance regulator (CFTR) modulators can rescue this severe phenotype ( Sun *et al*., 2019). By contrast, the CF mouse is widely used, with >14 different distinct models available ( Guilbault *et al.*, 2007). However, and despite the widespread availability of these murine CF models, their usefulness for studying chronic airway infection is limited. CF mice do not develop spontaneous airway infections ( Bayes *et al*., 2016; Cash *et al*., 1979; van Heeckeren *et al*., 2006), and artificially-induced infections of the airways are rapidly cleared if the inoculated microbial species are not immobilised using agar/agarose/alginate beads ( Chattoraj *et al*., 2010; Hoffmann *et al*., 2005; Moser *et al*., 2009; Munder *et al*., 2011). As with CF ferrets, this prevents the long-term study of polymicrobial communities and interspecies interactions.

Due to inherent limitations in the way in which the data is reported, the exact number of animals used for research into CF airway infections is difficult to ascertain. Yet a systematic review of the literature up to 2015 has found 12,304 publications discussing the use of CF animal models. Of those that are primary research articles (799 publications), 636 report the use of CF mice, rats, pigs, ferrets or zebrafish (with the remaining publications not reporting the use of any genetic CF models) ( Leenaars *et al*., 2020). Given that this literature review only accounts for publications up to 2015, and given the recent surge of interest into understanding the polymicrobial communities associated with CF airway infections, the number of CF animals used in research will have certainly increased over the last six years. Despite the importance of biofilms in CF pathology, and their well-known link with increased patient mortality rates, there is currently no suitable *in vivo* model for the study of polymicrobial biofilm communities in the context of CF. Furthermore, none of the aforementioned 636 studies report the use of CF models to study polymicrobial biofilm communities. Practical limitations when sampling the airway microbiota in infected animal models mean that the animals must be sacrificed and their lungs excised before histopathological examination. Not only are there significant ethical implications associated with these approaches, but the longitudinal/long-term study of microbial populations becomes impractical. More recently, *ex vivo* models of CF have redressed some of these issues, enabling the study of microbial biofilm lifestyles reminiscent of those observed *in situ* ( Harrington *et al*., 2020; Sweeney *et al*., 2021). However, owing to the intense competition observed among microbial species, existing *ex vivo* models do not yet permit the study of more than one microbial species at a time. This is a significant experimental limitation when studying long-term polymicrobial infection scenarios such as CF. Hence, the *in vitro* model we describe here provides a novel tool for the study of mixed-species biofilms that is not currently possible using existing *in vivo* CF models.

The primary limitations of *in vitro* infection models, compared with *in vivo* models, is a lack of host cells and a functional immune system (which may contribute towards clearing microbial infections *in situ*). However, it should be noted that impaired immune clearance of microbes in an inherent feature of CF, which somewhat mitigates the lack of a functional immune system in *in vitro* models ( Cohen and Prince, 2012; Bonfield and Chmiel, 2017). Although *in vitro* models do not capture all aspects of human disease pathophysiology, they do provide an ideal tool for studying interspecies interactions between microbes ( O'Brien and Welch, 2019b). Emerging evidence suggests that chemical, not spatiotemporal, factors have the greatest impact on driving changes in microbial lifestyles ( Lopes *et al*., 2017; Quinn *et al*., 2015). Hence, the defined and experimentally-perturbable nature of *in vitro* models are an attractive option for studying microbial behaviour in a reductive manner. Furthermore, the development of artificial sputum medium (ASM), closely mimicking the nutritional composition of CF airway secretions, provides an unparalleled opportunity for the study of polymicrobial populations under conditions that chemically recapitulate the CF microenvironment ( Haley *et al*., 2012; Sousa *et al*., 2018; Sriramulu *et al*., 2005; Turner *et al*., 2015; Frapwell *et al*., 2018). However, simply mixing CF airway-associated species together in ASM and hoping for the best is not a recipe for success. This is because the co-cultures display compositional instability, and the initially diverse community rapidly become dominated by just one or a few species. To remedy this, we developed an *in vitro* model of CF ( O'Brien and Welch, 2019a). Importantly, our model permits planktonic microbial communities of three distinctly different species associated with CF airway infections (PA, *Staphylococcus aureus* (SA) and *Candida albicans* (CA)) to be maintained, indefinitely, as a stable steady-state community. In this report we build upon our existing model and describe a simple, yet versatile, method of culturing polymicrobial biofilms on different solid substrata.

The model described in this work permits the simultaneous cultivation of steady-state planktonic and biofilm communities, allowing for direct comparisons to be made between these two modes of microbial growth. In principle, any combination of microbial species associated with CF airway infections could be cultured using the setup. As such, not only does our system reduce the need for *in vivo* CF infection models for studying microbe-microbe interactions; it enables the real-time, longitudinal study of polymicrobial communities in an experimentally reproducible, controlled setup. It has not escaped our notice that this model system is also well-suited for the road-testing of interventions aimed at preventing the formation of biofilms in chronic CF airway infections.

## Methods

### Methods for the model development

Microbial strains

All microbial strains used in this work are shown in Table 1 *.* Strains were routinely cultured in lysogeny broth (LB) (Formedium) on a 40 cm diameter rotating drum with mild aeration (0.5 rotations per second) at 37°C overnight.

**Table 1.** Microbial strains used in this study.

Strain	Description	Reference
PAO1	*Pseudomonas aeruginosa*, spontaneous chloramphenicol-resistant derivative. Used worldwide as a laboratory reference strain (isolated Melbourne, 1954)	( Holloway, 1955)
ATCC 25923	*Staphylococcus aureus* Rosenbach (ATCC ^®^25923D-5™), methicillin sensitive clinical isolate. Laboratory reference strain lacking recombinases and *mecA* (isolated Seattle, 1945)	( Treangen *et al*., 2014)
SC5314	*Candida albicans,* clinical isolate commonly used as a wild-type laboratory reference strain (isolated New York, 1980’s)	( Gillum *et al*., 1984)

Continuous-flow culture vessel and biofilm container

**Figure 1.**
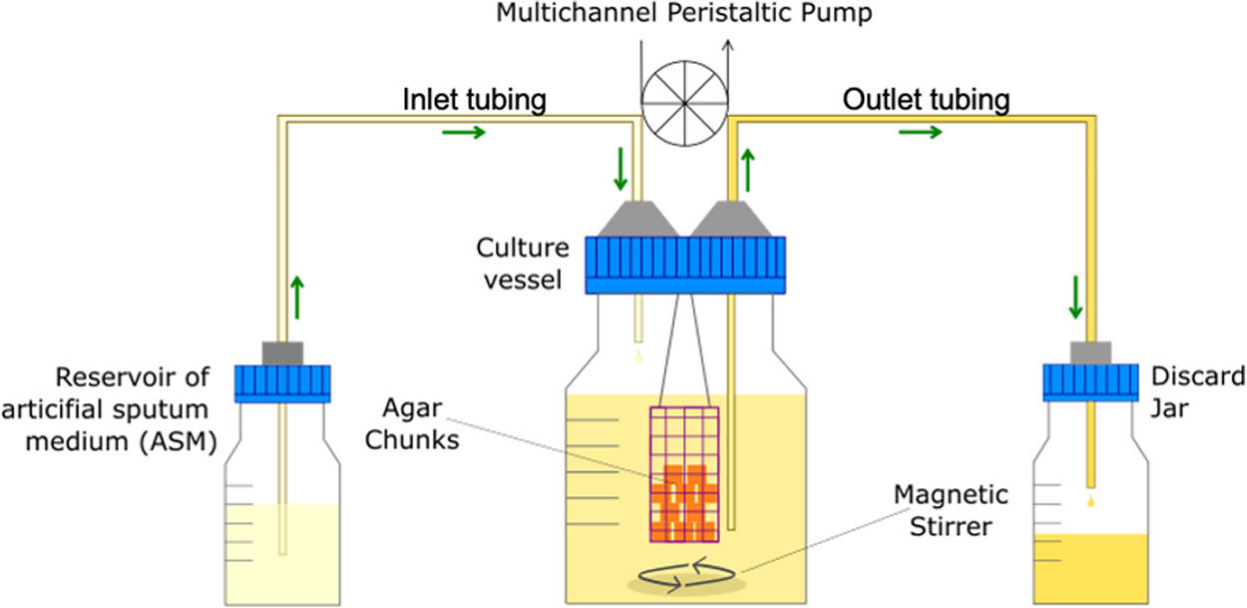
Schematic diagram of the continuous-flow culture vessel containing biofilm container. The main culture vessel (centre) is a 100 mL Duran bottle fitted with a 4-port HPLC GL80 screwcap lid, containing four sealable inlet/outlet ports from which the biofilm container is suspended. A multichannel peristaltic pump delivers fresh media (ASM) into the culture vessel from a reservoir (left), and also removes waste culture into a discard jar (right) at the same rate of flow ( *Q*). Arrows show the direction of media flow. The culture vessel and media reservoirs are incubated at 37°C and the contents are kept homogenous through gentle stirring (100 rpm). The value of *Q* depends on the microbial species being cultured within the vessel.

**Figure 2.**
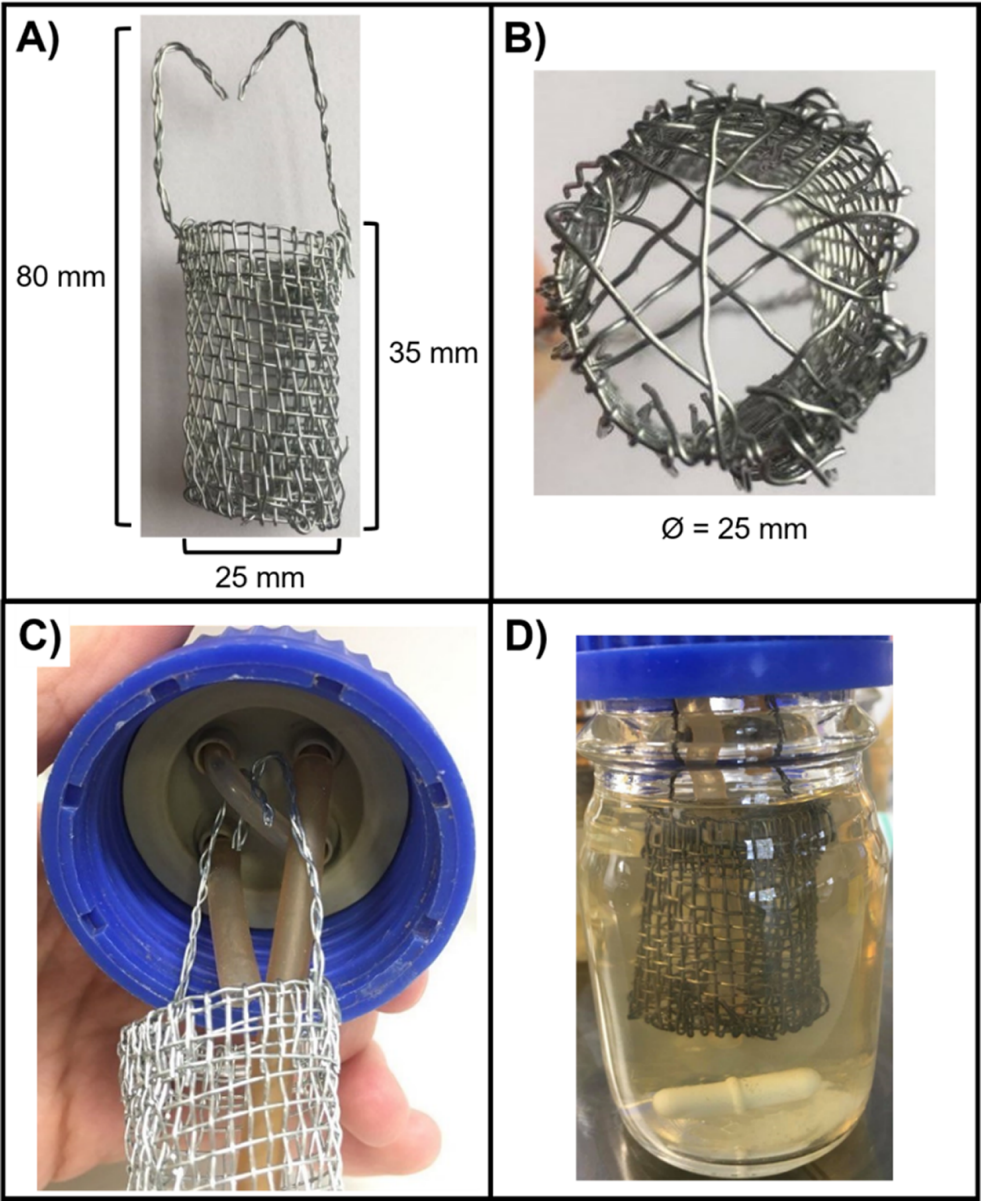
Container for culturing biofilms under continuous-flow conditions. The biofilm container constructed from stainless steel gauze for this study. Biofilm containers consisted of a cylinder, 35 mm in length and 25 mm in diameter (Ø), connected to two stainless steel arms (45 mm in length), which suspend the container from the HPLC screw-port lid. (A) Side view of the container with dimensions. (B) Bottom view of the container. Single strands of stainless-steel wire were threaded across the bottom of the container to form a mesh and prevent the biofilm substratum from falling through. A small gap (~3 mm Ø) was also included for the outlet tube to be threaded through to allow the removal of culture media during incubation. (C) View of the 4-port HPLC screw cap lid. The left port is the media inlet, the right port is the media outlet and the two remaining ports are threaded with a single piece of rubber tubing (1.5 mm bore) to hold the arms of the mesh container. (D) The biofilm container *in situ* in the assembled setup. Note that the container is completely submerged in the growth medium but is separated from the magnetic stir bar to prevent interference with the continual stirring of the culture vessel.

Artificial sputum medium (ASM) was used as the main growth medium for all experiments and was prepared as previously described ( O'Brien and Welch, 2019a). The continuous-flow culture system has also been previously described ( O'Brien and Welch, 2019a). Both are described in more detail in the protocol below. Briefly, the culture vessel consists of a 100 mL Duran flask, fitted with an assembled 4-port HPLC GL80 screw cap (Duran). A 24-channel IPC ISM934C standard-speed digital peristaltic pump (Ismatec) was used to deliver sterile ASM from a media reservoir at a defined flowrate ( *Q*) through 1.5 mm bore sterilin silicon tubing (Fisher Scientific) to the culture vessel. A different channel of the same pump was used to remove waste culture into a discard jar at the same flowrate ( Figure 1). Biofilms were allowed to develop in the continuous-flow culture vessel on two types of solid substratum; agar chunks and *ex vivo* porcine lung tissue (EVPL) sections, which have been previously reported to closely mimic the airway surface environment ( Harrison *et al*., 2014; Harrison and Diggle, 2016; Sweeney *et al*., 2021; Harrington *et al*., 2020). These substrata were suspended in bespoke cylindrical biofilm containers (25 mm diameter × 35 mm length) constructed from stainless steel wire gauze (Fisher Scientific) ( Figure 2A,B). Containers were then suspended in the culture vessel hanging from a piece of silicon tubing threaded through two of the unused HPLC ports ( Figure 2C). The entire culture system was maintained at 37°C and the liquid contents of the vessel were kept homogenous by stirring (100 rpm) using a round magnetic stir bar (length 20 mm, diameter 8 mm).

Preparation of biofilm substratum

**Figure 3.**
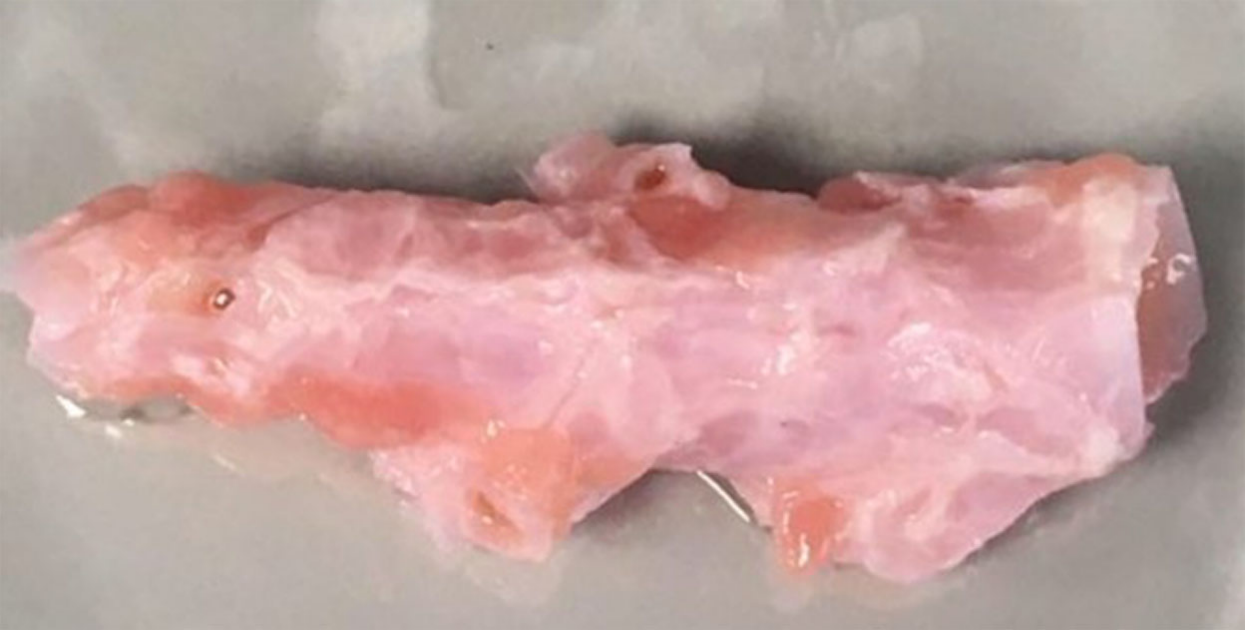
*Ex vivo* porcine bronchiole. A single bronchiole excised from porcine lung tissue after cleaning and a single wash in DMEM/RPMI solution. All alveolar and vascular tissue was removed using a razor blade and dissection scissors. After cleaning, bronchiole tissue was cut into 5 mm × 5 mm squares for use as solid substratum to promote biofilm growth.

Agar plates (2.5% w/v agar in milliQ water) were poured to a depth of 5 mm. Using a sterile number 21 scalpel and a set of sterile 12.7 cm curved forceps, cubes of approximately 5 mm x 5 mm were cut from the plate and transferred into the sterile biofilm container. Biofilm work using EVPL sections was performed in collaboration with Dr Freya Harrison at the University of Warwick, using previously described methods ( Harrison and Diggle, 2016). Briefly, fresh pig lungs were collected from the butcher (John Taylor, Earlsdon, UK) and processed within the hour. To remove surface contaminants, a 25 cm palette knife was heated until red-hot using a Bunsen burner and briefly tapped (< 1 s) on the area to be dissected. Working with aseptic technique, a sterile razor blade was used to excise the bronchiole and remove all alveolar tissue; a cleaned bronchiole can be seen in Figure 3 *.* Bronchioles were washed in Dulbecco's Modified Eagle Medium (DMEM)/Roswell Park Memorial Institute 1640 Medium (RPMI) solution (50:50 ratio, 40 mL) and cut into 5 mm wide strips with sterile dissection scissors. The strips were washed in DMEM/RPMI and cut into 5 mm × 5 mm squares. EVPL sections were washed again in DMEM/RPMI, then transferred to a petri dish containing 40 mL ASM and irradiated in UV lightbox for 5 min before being aseptically transferred to the biofilm container [Full dissection methods for the production of EVPL tissue sections are also demonstrated in an open access video protocol ( Harrington *et al*., 2021)].

Culture vessel inoculation and incubation

Overnight cultures of the microbial strains (grown in LB as described above) were washed three times in phosphate-buffered saline (PBS) prior to inoculating the culture vessel. Pre-warmed ASM (37°C, 100 mL) was added to the culture vessel and inoculated with the required combination of microbial species. The optical density (at 600 nm; OD _600 nm_) of the washed microbial cultures was then measured using a spectrophotometer (Eppendorf BioSpectrometer kinetic) and each species was introduced into the culture vessel to achieve a starting OD _600 nm_ of 0.05. The culture vessel was then incubated for three hours with stirring prior to starting the flow of medium ( *Q* = 145 μL min ^−1^).

Sample collection

For characterisation of biofilm populations, pieces of substratum were aseptically removed at the indicated times and transferred to 500 μL PBS in 24-well microtiter plates. Loosely attached planktonic cells were removed from the substratum by briefly swirling the plates. The substratum was then transferred to a second well in the same plate and washed once more. Finally, after a third wash, the samples were transferred into 2 mL bead beating tubes containing 1 mL PBS and eighteen metal beads (2.38 mm diameter, Qiagen). The tubes were them agitated in a FastPrep-24 5G benchtop homogeniser (MP Biomedicals) for 40 s at 4 m s ^−1^ to liberate the cells. CFU mL ^−1^ counts were performed as described below. Three separate substratum pieces were sampled at each time point (yielding three biological replicates per timepoint, per independent biological experiment). Samples of the planktonic culture (1 mL) were removed directly from the culture vessel using a sterile serological pipette. Independent biological experiments were then performed over separate independent weeks using fresh microbial cultures and pieces of biofilm substratum without any deviation from the methods detailed in this article.

CFU mL ^−1^ enumeration

Colony forming units (CFU) were determined using the single plate-serial dilution spotting method, as described previously ( Thomas *et al*., 2015). Briefly, 10-fold serial dilutions of the microbial cultures were made in sterile PBS and 20 μL of each dilution was spotted (approximately 2 μL per drop) onto the appropriate selective agar. Three types of selective agar were used to enumerate microbial cell counts of the different species present in a single sample: PA was isolated using *Pseudomonas* isolation agar (PIA, Oxoid), SA was isolated using mannitol salt agar (MSA, Oxoid) and CA was isolated onto BiGGY agar (Oxoid). When enumerating cell counts from the polymicrobial cultures, the agar plates used to isolate PA and SA were further supplemented with 5 μg mL ^−1^ itraconazole to inhibit the growth of CA. The different selective media only permit the growth of a single microbial species of interest and inhibit the growth of the other species present. This enables CFU mL ^−1^ counts for each species in the polymicrobial culture to be determined with confidence [note that when studying other co-cultures containing other combinations of microbial species, different types of selective media may be required for the selective enumeration of other species]. All plates were incubated at 37°C. PIA and MSA plates were incubated overnight (16 h) and BiGGY agar plates were incubated for 24 h. Three independent samples of biofilm substratum from the same culture vessel were removed per timepoint (corresponding to three biological replicates). For each biological replicate, three independent serial dilutions were made and plated out for CFU enumeration (constituting three technical replicates per each piece of substratum sampled), non-blinded CFU mL ^−1^ counts were then recorded as the average of the technical replicates. Two independent samples of culture supernatant were removed from each culture vessel per timepoint (corresponding to two technical replicates) to enumerate planktonic CFU mL ^−1^ counts of each species.

**Figure 4.**
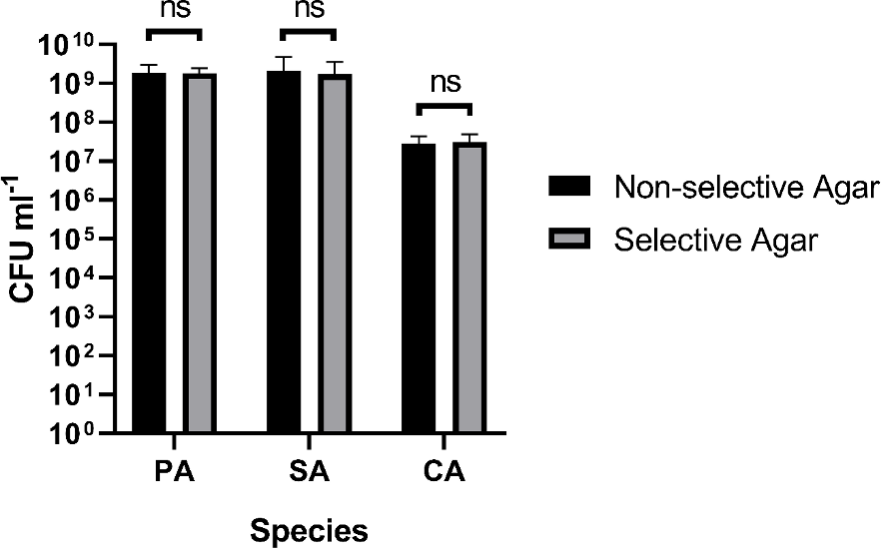
Comparison of cell titres on selective and non-selective agar. The figure shows the viable cell counts (expressed as CFU mL ^−1^) of overnight single-species cultures of *P. aeruginosa* PAO1 (PA), *S. aureus* 25923 (SA) and *C. albicans* SC5314 (CA) plated on non-selective agar media (black bars) and selective agar media (grey bars). Data represent the mean ± standard deviation from two technical replicates collected from three independent biological experiments. *P* > 0.05 is considered as not significantly different (ns).

We tested whether there was any significant difference in cell counts determined on non-selective *vs* selective agar, and there was not ( Figure 4 and underlying data ( O'Brien *et al*., 2021)). To test this, we performed serial dilutions of overnight microbial cultures (routinely grown in LB as described above) and plated the same dilution series onto LB-agar (non-selective) and the appropriate selective agar for the cultured species [see notes above]. Plates were then incubated in the same static incubator at 37°C. Plates containing PA or SA incubated for 16 h and plates containing CA incubated for 24 h (as described above). Two independent serial dilutions of the same overnight culture were prepared and plated in parallel from the same overnight culture (constituting two technical replicates) and three independent biological replicates for each microbial species were performed across separate days using fresh microbial cultures and freshly prepared agar plates.

Statistical analysis

All biofilm data are represented as the mean ± standard deviation (SD) of three separate biological replicates (collected simultaneously from the same culture vessel at each point of sampling) per timepoint across three independent biological experiments (conducted across different weeks) that were performed using fresh: ASM, microbial cultures and pieces of biofilm substrata [note that experiments using agar chunks or EVPL sections were performed independently of one another]. All planktonic data are represented as the mean ± standard deviation (SD) of two technical replicates (collected simultaneously from the same culture vessel) per timepoint across three independent biological experiments. Planktonic and biofilm CFU mL ^−1^ counts were performed in parallel from the same culture vessel. All statistical analysis was performed using GraphPad Prism version 8.2.0, with *P* < 0.05 being considered statistically significant [statistical analysis can also be performed using R/ Python packages stats/ SciPy, respectively]. Paired group two-tailed t-tests were used to analyse: changes in CFU mL ^−1^ counts of the different species present in single- or mixed-species biofilm samples collected at T = 24 h or 96 h; differences in CFU mL ^−1^ counts of the microbial species present on either agar chunks or EVPL sections; and for comparisons of cell viability on selective and non-selective media. Changes in planktonic CFU mL ^−1^ counts across the timepoints for each experiment were analysed using one-way repeated measures ANOVA. Differences in planktonic CFU mL ^−1^ counts in cultures containing the different biofilm substrata across the timepoints for each experiment were analysed using two-way repeated measures ANOVA.

### Protocol for the use of this model

Here we describe step-by-step the procedure used to prepare ASM and to set up/inoculate the continuous-flow culture vessel for the study of polymicrobial biofilm and planktonic communities. Reagents and equipment used in this study are listed in Table 2 and Table 3, respectively.

1. *Step 1* Assembly of the biofilm containers. This can be done any time prior to making ASM. A wire “bucket” was crafted by hand from stainless steel wire gauze (Fischer Scientific). Figure 2 shows an image of a completed container and dimensions. Wire cutters are used to trim a mesh of stainless-steel gauze to a rectangle approximately 150 mm x 40 mm. The gauze was then carefully rolled into a cylinder with an approximate diameter of 25 mm. The protruding cut ends of the wire mesh on the side of the cylinder were threaded through the adjacent mesh to secure the side of the cylinder in place. The height of the bucket was then adjusted to 35 mm *via* trimming with the wire cutters. Spare lengths of wire thread were then woven across the base of the container to generate a bucket-like structure ( Figure 2B). Note that we ensured that there was a hole in the bottom of the bucket large enough to fit the out-flow tubing (~ 3mm diameter) ( *Step 6*). To keep the stainless-steel container from interfering with the magnetic stirrer, and to allow easy removal of substratum material during cultivation, we suspended the bucket *via* two wire “arms” from a loop of silicone tubing threaded through two unused ports of the HPLC screw cap lid ( Figure 2C). The wire arms were made by entwining three strands of stainless-steel wire and threading these through the mesh at the top of the bucket. The protruding portion of the arms were bent to allow the bucket assembly to be draped over the silicone tubing support loop and trimmed in length to 45 mm. The correct length of these container arms is important to ensure that the bucket is completely submerged in the culture medium throughout incubation ( Figure 2D).
2. *Step 2* Preparation of ASM (day 1). Add 5 g of Type II mucin from porcine stomach to 250 mL phosphate buffered saline (pH 7.4) and leave to dissolve overnight with stirring (~400 rpm) at 4°C. On the same day, add 4 g of deoxyribonucleic acid from salmon sperm to 250 mL autoclaved milliQ water and leave to dissolve overnight in a shaking water bath (200 rpm, 30°C). While the mucin/DNA is dissolving, prepare the amino acid and salt/buffer stock solutions (50 mL of each) according to Table 4. Most amino acid stock solutions can be kept for one month in the dark at 4°C. However, stock solutions of tyrosine, threonine, cysteine, phenylalanine and histidine must be made freshly for each batch of ASM.
3. *Step 3* Preparation of ASM (day 2). Add ammonium chloride (0.124 g), potassium chloride (1.116 g), sodium chloride (3.032 g) and 3-( *N*-morpholino) propanesulfonic acid (MOPS, 2.092 g) to a 1 L beaker and dissolve in 250 mL milliQ water with gentle stirring (50 rpm). Then add the remaining salts and amino acids from the pre-made stock solutions as shown in Table 4. Next, combine the dissolved DNA and mucin solutions and add to the beaker with gentle mixing (50 rpm) for 5 min, or until the solution is homogenous. Adjust to pH 6.8 with 0.1 M acetic acid or 0.1 M potassium hydroxide. Following this, prepare the remaining media solutions and add them to the beaker according to Table 5 alongside 5 mL egg yolk emulsion. Once fully homogenous, adjust the total volume to 1 L with milliQ water. The ASM is then filter sterilised using a 1 L disposable Stericup filter unit (0.22 μM pore size) attached to a large diaphragm vacuum pump. Note that the filtering process is slow and may take 2–3 days to complete. Half-way through filtering we recommend carefully decanting (into a clean beaker) the unfiltered ASM from the Stericup unit and rinsing the top of the filter membrane with several mL of sterile PBS (that is then discarded) and then continuing with the filtration to speed up this process.
4. *Step 4* Assembly of continuous-flow culture vessel (day 3). While the ASM is being filter-sterilised, assemble the continuous-flow culture vessel as outlined below with inclusion of the biofilm bucket constructed in *Step 1.* The continuous-flow culture vessel consists of a 100 mL Duran flask fitted with an assembled GL-80 4-port HPLC screw cap (Duran). Two lengths of 1.5 mm bore sterlin silicone tubing (Fisher Scientific) were fed into two of the HPLC ports to act as an inlet/outlet (respectively) for the medium. To prevent contamination of the media reservoir with motile bacteria, ensure that only the outlet tube is in contact with the culture medium when the vessel is filled with ASM. A short piece of silicone tubing was threaded through the two-remaining unused HPLC ports (diagonal from one another) and secured tightly before sealing any gaps around the HPLC ports with parafilm. The biofilm bucket (assembled in *Step 1*) was hooked onto the suspended tubing and, using forceps, the outlet tube was gently pulled through the meshed hole in the base of the bucket (see *step 1*). A clean magnetic stir bar was placed in the culture vessel and the fully-assembled lid was fitted. Finally, the inlet tubing was wrapped in aluminium foil and the entire assembly was autoclaved. The setup was allowed to completely dry overnight (in a drying cabinet) before use.
5. *Step 5* Preparation of biofilm substratum (day 3). (i) Using agar chunks as a solid substratum for biofilm formation, prepare the substratum by suspending 2.5 g of agar in 100 mL milliQ water. Autoclave. Once cooled, but still warm (~65°C), pour the agar to a depth of 5 mm in a sterile 90 mm petri dish. Once set, the agar plates can be wrapped in parafilm and kept at 4°C until the day of use. (ii) *Ex vivo* porcine lung (EVPL) tissue sections can also be used as an alternative substratum for biofilm formation. Please refer to *Step 7* (day 4) for the protocol detailing the inclusion of EVPL tissue sections in the model.
6. *Step 6* Preparation of overnight microbial cultures (day 3). Place a nichrome 5 μL microbial inoculation loop into the blue flame of a Bunsen burner for 5 s. Allow the loop to cool then pick a single microbial colony from an agar plate and use to inoculate 10 mL of LB in a sterile 30 mL universal tube. Repeat for all microbial strains to be inoculated into the culture vessel, then place the tubes on a rotating drum (200 rpm) and incubate at 37°C overnight.
7. *Step 7* Preparation of EVPL biofilm substratum (day 4). This work was conducted in collaboration with Dr Freya Harrison at the University of Warwick following their previously published protocol for the preparation of EVPL sections ( Harrison *et al.*, 2014) Prior to culture vessel assembly and inoculation (see steps 8-9 below), fresh pig lungs were collected from the butchers and processed within the hour. To remove surface contaminants, a 25 cm palette knife was heated using a Bunsen burner and briefly tapped (< 1 s) on the area to be dissected. A sterile razor blade was used to excise the bronchiole and remove all alveolar tissue. Bronchioles were then washed in DMEM/RPMI solution (40 mL, 50:50 ratio) and cut into 5 mm wide strips with sterile dissection scissors. The strips were washed in DMEM/RPMI and cut into 5 mm × 5 mm squares. EVPL sections were washed again in DMEM/RPMI, then transferred to a petri dish containing 40 mL ASM and irradiated in UV lightbox for 5 min before being aseptically transferred to the biofilm container (as in *Step 8).*
8. *Step 8* Assembly of the culture vessel (day 4). Working in a microbiological safety cabinet, cut the agar into 5 mm × 5 mm cubes using sterile (autoclaved) scalpel/forceps and aseptically transfer these into the wire mesh container so that they sit loose (not stacked) at the bottom of the bucket. The number of substratum pieces used for an experiment may differ, depending on the type of experiment, length of incubation and number of time points to be sampled. In the current work, we simply report the microbial composition of polymicrobial biofilms at two time points (T = 24 h and 96 h). Hence, only six pieces of substratum were incubated in the culture vessel (three for each time point). However, we note that each biofilm container can easily hold 30+ pieces of substratum. Next, aseptically transfer 100 mL of fresh ASM to the culture vessel and secure the lid, ensuring that the biofilm container does not interfere with the magnetic stir bar and is fully submerged in the ASM. The inlet tubing was carefully unwrapped from the foil and placed into the remaining ASM (forming the media reservoir) with the top sealed well with sterile parafilm to prevent contamination. Finally, place the end of the outlet tubing into a discard jar and connect the inlet/outlet tubing to a peristaltic pump. Figure 1 shows a schematic diagram of the assembled continuous-flow culture vessel. The entire system can then be primed with media. The setup was pre-warmed at 37°C prior to microbial inoculation.
9. *Step 9* Culture vessel inoculation and incubation (day 4). In this report we describe the formation of polymicrobial biofilms of *Pseudomonas aeruginosa* (PA), *Staphylococcus aureus* (SA) and *Candida albicans* (CA), although in principle, any combination of microbial species could be used for co-culture. Overnight cultures (prepared on day 3) of each species were washed three times in sterile PBS prior to inoculation. Once washed, the optical density (OD _600_) of the cultures was measured and each species was introduced into the culture vessel to achieve a starting OD _600_ of 0.05. The culture vessel was incubated at 37°C, with 100 rpm stirring using a round PTFE magnetic stir bar (8 mm diameter × 20 mm length), for three hours prior to starting the flow of medium. For the current study, a continuous flow rate of 145 μL min ^−1^ was applied for the remaining period of incubation (96 h). From our previous study ( O'Brien and Welch, 2019a), we note that the flow rate may need to be experimentally optimised if the goal is to maintain steady-state cultures of other species or strains.
10. *Step 10* Sample removal and viable cell counts (Day 4–9). At the desired time point(s) (in this study, T = 24 h and 96 h for the biofilms and T = 24, 48, 72 and 96 h for the planktonic samples) remove 1 mL aliquots of the liquid culture using a sterile serological pipette, or pieces of the biofilm substratum from the biofilm bucket, as necessary. For enumeration of colony forming units (CFU mL ^−1^) from the biofilm samples, remove pieces of solid substratum using sterile forceps, taking care to flame and cool the forceps between the collection of individual samples. Pieces of the substratum were aseptically transferred to 500 μL sterile PBS in a 24-well plate. To remove any loosely attached planktonic cells, gently swirl the plates for approximately 5 s then transfer the substratum to another 500 μL of fresh PBS. Repeat the process two more times. A washed piece of substratum was then transferred to a 2 mL bead beating tube containing 1 mL PBS and eighteen 2.38 mm diameter metal beads (Qiagen). The sample was homogenised in a Fast-Prep 5G benchtop homogeniser (MP biomedicals) for 40 s at 4 m s ^−1^. Using a pipette, and taking care to avoid any solid pieces of biofilm substratum, the cell supernatant was removed and CFU mL ^−1^ counts were determined as described in the Methods section above.

**Table 2.** Reagents used in this study.

Reagent	Supplier [Catalogue Number]
Mucin from porcine stomach (Type II)	Merck [M2378]
Deoxyribonucleic acid from salmon sperm	Merck [31149]
Phosphate buffered saline	Oxoid [BR0014G]
*L*-serine	Merck [S4500]
*L*-glutamic acid hydrochloride	Merck [G2128]
*L*-proline	Merck [P8865]
*L*-glycine	Merck [G7126]
*L*-alanine	Merck [A7627]
*L*-valine	Merck [V0500]
*L*-methionine	Merck [M9625]
*L*-isoleucine	Merck [I2752]
*L*-leucine	Merck [L8000]
*L*-ornithine hydrochloride	Merck [W419001]
*L*-lysine hydrochloride	Merck [W384712]
*L*-arginine monohydrochloride	Merck [A5131]
*L*-tryptophan	Merck [T0254]
*L*-asparagine monohydrate	Merck [A8381]
*L*-tyrosine	Merck [T3754]
*L*-threonine	Merck [T8625]
*L*-cystine dihydrochloride	Merck [C6727]
*L*-phenylalanine	Merck [P2126]
*L*-histidine monohydrochloride monohydrate	Merck [H8125]
Sodium phosphate monobasic anhydrous	Fisher Scientific [7558-80-7]
Sodium phosphate dibasic anhydrous	Fisher Scientific [7558-79-4]
Potassium nitrate	Fisher Scientific [7757-79-1]
Potassium sulfate	Fisher Scientific [7778-80-5]
Ammonium chloride	Merck [12125-02-9]
Sodium chloride	Fisher Scientific [7647-14-5]
Potassium chloride	Fisher Scientific [7447-40-7]
MOPS [3-( *N*-morpholino)-propanesulfonic acid]	Melford [1132-61-2]
*D*-(+)-glucose (dextrose)	Merck [G7021]
*L*-(+)-lactic acid	Merck [L1750]
Calcium chloride dihydrate	Merck [10035-04-8]
Magnesium chloride hexahydrate	Merck [7791-18-6]
Iron (II) sulfate heptahydrate	Merck [7782-63-0]
*N*-acetyl- *D*-glucosamine	Merck [A8625]
Egg yolk emulsion	Millipore [17148]
Pseudomonas isolation agar	Oxoid [CM0559]
Mannitol salt agar	Oxoid [CM0085]
BiGGY-agar	Oxoid [CM0589]
Agar	Formedium [009002-18-0]
Lysogeny broth	Formedium [LBX0102]
Dulbecco's Modified Eagle Medium	Merck [D5030]
Roswell Park Memorial Institute 1640 Medium	Merck [R8758]

**Table 3.** Equipment used in this study.

Equipment	Supplier [Catalogue Number]
100 mL Duran Flask	Merck [Z232076]
4-port HPLC GL80 Screw Cap	Fisher Scientific [10583913]
24-channel IPC ISM934C Standard-speed Digital Peristaltic Pump	Cole-Parmer [WZ-78001-42]
1.5 mm Bore Sterilin Silicon Tubing	ThermoFisher [TSR0150150P]
Stainless Steel Wire Gauze	Fisher Scientific [12958950]
Velp 6-position Multiposition Digital Stirrer	Cole-Parmer [F203A0179]
Round White PTFE Magnetic Stir Bar, Length 20 mm x Diameter 8 mm	Merck [HS120548]
Swann-Morton Number 21 Carbon Steel Scalpel Blade	Fisher Scientific [11778363]
Epredia Shandon 12.7 cm Cartilage Fine Point Curved Thumb Forceps	Fisher Scientific [15307805]
Epredia Shandon 16.5 cm Straight Dissecting Scissors (Sharp Ended)	Fisher Scientific [15202290]
25 cm Eisco Palette Knife Spatula	Fisher Scientific [S80827]
Azpack Carbon Steel Razor Blades	Fisher Scientific [11904325]
Microspec 5 **μ**L Nichrome Microbial inoculation Loop	Fisher Scientific [15712175]
30 mL Polystyrene Universal Tube (Sterile)	Starlab [E1412-3011]
Eppendorf BioSpectrometer Kinetic	Eppendorf [6136000819]
2.38 mm Diameter Metal Beads	Qiagen [13118-400]
2 mL PowerBead Beating Tubes	Qiagen [13116-50]
FastPrep-24 5G benchtop homogeniser	MP Biomedicals [116005500]
Stericup Quick Release-GP Sterile Vacuum Filtration System	Merck [S2GPU10RE]
Nunc Cell-Culture Treated 24-well Plate	ThermoFisher [142475]
Sterile 90 mm × 15 mm Petri Dishes	Merk [Z717223]
Bemis Parafilm M Laboratory Wrapping	Fisher Scientific [HS234526B]

**Table 4.** Amino acid and salt stock solutions and volumes needed to make 1 L ASM.

Chemical	Mass to add (g)	Stock volume (mL)	Stock conc (M)	Stock to add to beaker (mL)	Final Conc (mM)	Notes
*L*-serine	0.525	50	0.1	14.46	1.446	-
*L*-glutamic acid hydrochloride	0.918	50	0.1	15.492	1.549	-
*L*-proline	0.576	50	0.1	16.612	1.661	-
*L*-glycine	0.375	50	0.1	12.032	1.203	-
*L*-alanine	0.445	50	0.1	17.8	1.78	-
*L*-valine	0.586	50	0.1	11.172	1.117	-
*L*-methionine	0.746	50	0.1	6.332	0.633	-
*L*-isoleucine	0.656	50	0.1	11.212	1.121	-
*L*-leucine	0.656	50	0.1	16.092	1.609	-
*L*-ornithine hydrochloride	0.843	50	0.1	6.76	0.676	-
*L*-lysine hydrochloride	0.913	50	0.1	21.28	2.128	-
*L*-arginine monohydrochloride	1.054	50	0.1	3.06	0.306	-
*L*-tryptophan	1.021	50	0.1	0.132	0.013	Prep in 0.2 M NaOH
*L*-asparagine monohydrate	0.666	50	0.1	8.272	0.827	Prep in 0.5 M NaOH
*L*-tyrosine	0.906	50	0.1	8.02	0.802	Prep in 1.0 M NaOH Make fresh
*L*-threonine	0.596	50	0.1	10.72	1.072	Make fresh
*L*-cystine dihydrochloride	0.788	50	0.1	1.6	0.16	Make fresh
*L*-phenylalanine	0.826	50	0.1	5.3	0.53	Make fresh
*L*-histidine monohydrochloride monohydrate	1.048	50	0.1	5.192	0.519	Make fresh
Sodium phosphate monobasic anhydrous	1.380	50	0.2	8.125	1.3	-
Sodium phosphate dibasic anhydrous	1.420	50	0.2	6.252	1.25	-
Potassium nitrate	5.056	50	1	0.348	0.348	-
Potassium sulfate	2.178	50	0.25	1.084	0.271	-

**Table 5.** Reagents to make 1 L ASM after addition of the mucin-DNA solution and pH adjustment to pH 6.8.

Chemical	Mass to add (g)	Stock volume (mL)	Stock conc (M)	Stock to add to beaker (mL)	Final Conc (mM)	Notes
*D*-(+)-glucose (dextrose)	4.504	25	1	1.2	3	-
*L*-(+)-lactic acid	2.252	25	1	9.3	9.3	pH stock to 7 with NaOH
Calcium chloride dihydrate	3.67535	25	1	1.754	1.754	-
Magnesium chloride hexahydrate	5.08275	25	1	0.606	0.606	-
Iron (II) sulfate heptahydrate	0.05	50	0.0036	1	0.0036	Make fresh
*N*-acetyl- *D*-glucosamine	1.383	25	0.25	1.2	0.3	-

## Results

### Characterisation of the biofilms formed in continuous flow conditions

(i) Agar as a solid substratum

**Figure 5.**
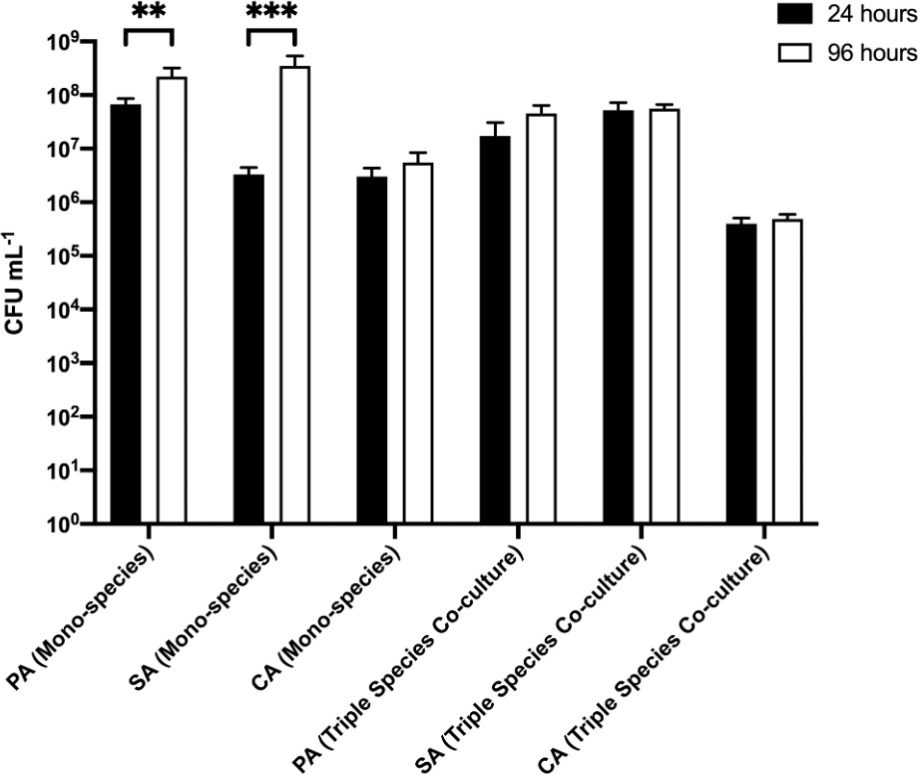
Cell titres derived from biofilms adhered to agar chunks. *P. aeruginosa* PAO1 (PA), *S. aureus* 25923 (SA) and *C. albicans* SC5314 (CA) cell counts (expressed as CFU mL ^-1^) adhered to 2.5% (w/v) agar chunks after incubation for 24 h (black bars) or 96 h (white bars) in the continuous-flow culture model. Asterisks indicate significant (** *P* < 0.01, *** *P* < 0.001) differences in CFU mL ^-1^ counts between the 24 h and 96 h time points. Data represent the mean ± standard deviation of three independent biological experiments in which three pieces of biofilm substratum were sampled per culture vessel per timepoint.

We first sought to discern changes in the microbial composition of single-species and polymicrobial biofilms grown on agar chunks as a substratum ( Figure 5 and underlying data ( O'Brien *et al*., 2021)). Viable cell counts were determined on selective agar plates after rinsing each 5 mm agar chunk (with attached biofilm) and resuspending the attached cells in 1 mL PBS. When examining the single-species biofilm populations, titres of attached SA and CA were similar (ca. 10 ^6^–10 ^7^ CFU mL ^−1^) after 24 h incubation, whereas titres of attached PA were >10-fold greater (at just under 10 ^8^ CFU mL ^−1^). However, over the next 72 h, PA titres increased only slightly (albeit significantly) to >10 ^8^ CFU mL ^−1^, and SA titres rose to around the same level (i.e. to a titre 2 logs greater than the titre at the 24 h time point). By contrast, CA titres remained essentially unchanged at the 96 h time point compared with the 24 h time point. These data suggest CA biofilms establish rapidly with little overall change in cell titres over time. By contrast, PA and SA biofilms also establish quickly, but continue to grow significantly between sampling points.

The situation was different in the polymicrobial cultures. Here, the titres of PA, SA, and CA present on the biofilm substrata removed sequentially from the same culture vessel remained essentially unchanged at the 24 h and 96 h sampling points (at around 10 ^7^ CFU mL ^−1^ for PA and SA, and around 10 ^5^ CFU mL ^−1^ for CA). These data suggest that growth in a polymicrobial culture constrains the population size of individual species in a biofilm.

(ii) EVPL tissue sections as a solid substratum

**Figure 6.**
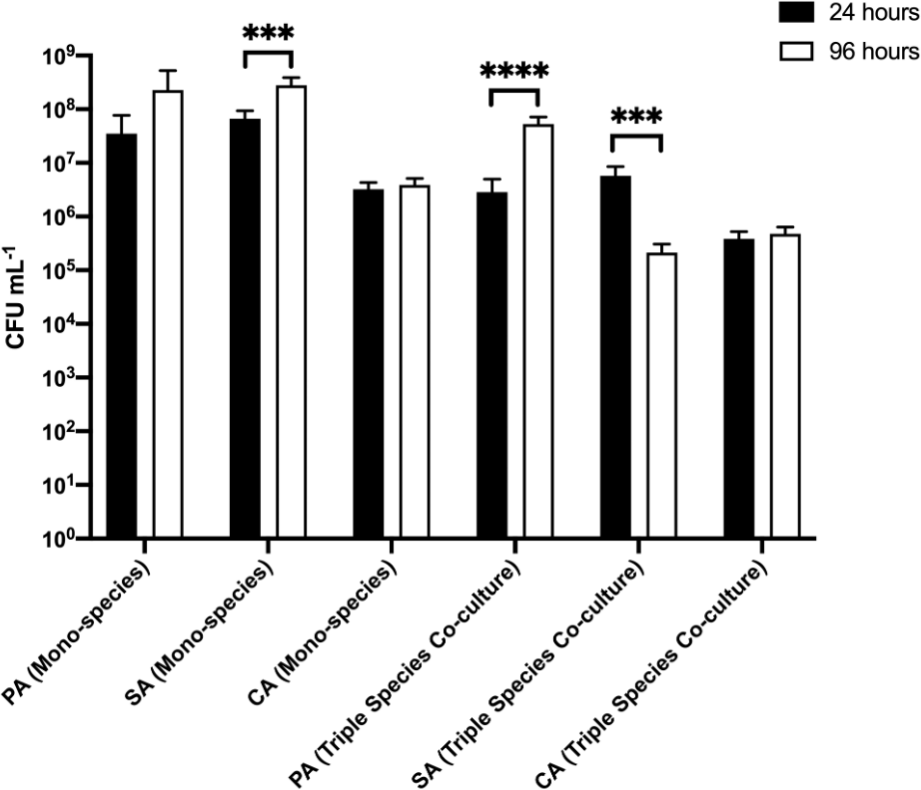
Cell titres derived from biofilms adhered to porcine bronchiole tissue. *P. aeruginosa* PAO1 (PA), *S. aureus* 25923 (SA) and *C. albicans* SC5314 (CA) cell counts (expressed as CFU mL ^−1^) adhered to sections of *ex vivo* porcine lung tissue after incubation for 24 h (black bars) or 96 h (white bars) in the continuous-flow culture model. Asterisks indicate significant (*** *P* < 0.001, **** *P* < 0.0001) differences in CFU mL ^−1^ counts between the 24 h and 96 h time points. Data represent the mean ± standard deviation of three independent biological experiments in which three pieces of biofilm substratum were sampled per culture vessel per timepoint.

We next examined biofilm formation on a different biofilm substratum, EVPL tissue (in place of agar chunks) ( Figure 6 and underlying data ( O'Brien *et al*., 2021)). No PA, SA or CA cells could be isolated from uninfected EVPL sections after any period of incubation. In the single-species biofilm populations, there was no appreciable difference in PA or SA titres adhered to the substratum at the initial point of sampling (10 ^7^–10 ^8^ CFU mL ^−1^ at 24 h). Adhered CA titres were consistently 10-fold lower (ca. 10 ^6^ CFU mL ^−1^) at the same sampling point. After a further 72 h growth, PA and SA titres increased to >10 ^8^ CFU mL ^−1^, whereas CA titres remained essentially unchanged compared with the titres measured at 24 h. These data indicate that the dynamics of SA biofilm formation varies on different substrata; SA clearly shows more robust initial colonisation of EVPL substrata compared with agar chunks.

In the triple-species polymicrobial culture, there was no statistically significant difference in adhered PA or SA titres at the 24 h sampling point (both species achieving 10 ^6^–10 ^7^ CFU mL ^−1^ at 24 h). We note that this is 10-fold lower than the PA and SA titres at the same time point in the corresponding biofilms from axenic cultures. Similarly, CA titres in the polymicrobial biofilms were also 10-fold lower than the CA titres in axenically-cultured biofilms. However, and whereas adhered CA titres in the polymicrobial biofilms remained unchanged over the following 72 h, PA titres displayed a marked increase (attaining ca. 10 ^8^ CFU mL ^−1^ by the 96 h time point) and SA titres displayed a marked decrease (falling to ca. 10 ^5^ CFU mL ^−1^) in adhered cell counts. We conclude that the population dynamics of each species can vary by orders of magnitude, depending on the nature of the biofilm substrata.

### Population structure of the planktonic fractions

**Figure 7.**
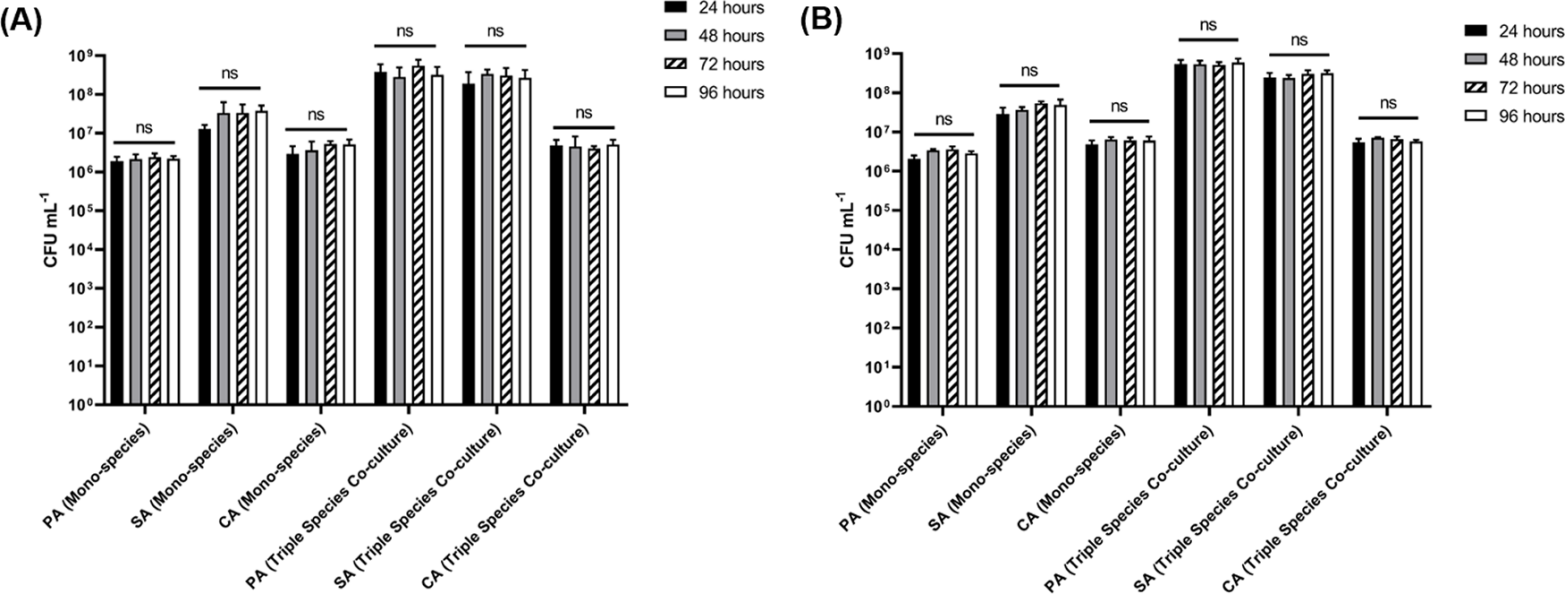
Cell titres of planktonic cultures. *P. aeruginosa* PAO1 (PA), *S. aureus* 25923 (SA) and *C. albicans* SC5314 (CA) cell counts (expressed as CFU mL ^−1^) in the planktonic fraction of single-species and polymicrobial cultures incubated in ASM in the continuous-flow model containing (A) agar chunks or (B) *ex vivo* porcine lung tissue sections as the solid substrata. Data represent the mean ± standard deviation of two technical replicates collected per timepoint from three independent biological experiments. *P* > 0.05 is considered as not significantly different (ns).

In parallel with the analysis of species titres in adhered biofilms, we also examined the corresponding titres of each species in the planktonic (i.e. non-attached) fraction ( Figure 7 and *underlying data* ( O'Brien *et al*., 2021)). Consistent with our previous findings ( O'Brien and Welch, 2019a), steady-state microbial communities were established by 24 h incubation. There was no appreciable change in CFU mL ^−1^ counts of any species cultured as part of an axenic or polymicrobial population across any point of sampling. Furthermore, there was no discernible difference in CFU mL ^−1^ counts of the planktonic communities grown in the culture vessel flasks containing agar chunks ( Figure 7A) or EVPL tissue sections ( Figure 7B). These data indicate that the population dynamics of the biofilms is distinct from the population dynamics of the surrounding planktonic culture.

## Discussion

Here we report a simple method for the robust and reproducible growth of single- and multi-species biofilms on different substrata. Our model system has been designed to mimic the nutritional environment associated with CF airways. We previously showed ( O'Brien and Welch, 2019a) that the model can maintain very stable steady-state polymicrobial populations of planktonic cells, even among species that would ordinarily outcompete one another during *ex situ* co-cultivation. In the current work, we extend these findings to show that polymicrobial biofilms can be similarly maintained. Remarkably, we also show that the biofilm composition is far more dynamic than that of the surrounding planktonic culture, and can vary substantially, even when the planktonic species profile is stable.

A major benefit of our model over existing *in vivo* approaches is that is inexpensive to set up and requires no specialist equipment or training to operate. Indeed, the only perceived barrier preventing wider uptake of the model is in preparation of the ASM culture medium. Mitigating, this generally requires only a few minutes of effort each day (once the stock solutions have been made up). The primary benefit of the model is that very stable polymicrobial communities can be rapidly established and maintained for extended periods of time, and that the planktonic and biofilm modes of growth can be studied concurrently in a single experiment. This enables the facile longitudinal study of polymicrobial communities *in vitro*, enabling experimental analyses that were previously not possible due to population instability. As such, the model allows researchers to explore previously inaccessible problems pertaining to microbial ecology, gene expression and metabolism in polymicrobial communities. For example, we are currently exploiting the system to examine how mixed-species biofilms such as those found in the CF airways form over time. We are also using the model to examine how the inclusion of different species or treatment with antimicrobial compounds affect the stability and dynamics of polymicrobial biofilms longitudinally.

Alongside the ethical benefits and accessibility of an *in vitro* model for microbial culture, the experimentally tuneable nature of our model system provides several inherent benefits over existing *in vivo* CF models ( O'Brien and Welch, 2019b). ASM is a chemically-defined synthetic growth medium that has been formulated to closely mimic the nutritional composition of CF airway secretions ( Kirchner *et al*., 2012; Palmer *et al*., 2005; Palmer *et al*., 2007; Turner *et al*., 2015; Cornforth *et al*., 2018). This allows subtle interspecies interactions to be quantified, in real-time, with great reproducibility. By contrast, the existing animal models display much greater variability, even within a single host species. Furthermore, culture conditions can be readily modified to enable the detailed study of how a particular variable (such as iron, for example) impinges upon the formation of polymicrobial biofilms ( Mashburn *et al*., 2005; Palmer *et al*., 2005). This level of environmental control is near impossible to achieve with *in vivo* infection models. We also note that the experimentally facile nature of our system should permit the study of biofilm formation in other clinically-relevant polymicrobial infection scenarios such as non-CF bronchiectasis, asthma, or chronic obstructive pulmonary disorders (COPDs). This can be accomplished through the simple expedient of appropriately modifying the nutritional composition of the culture medium.

The key finding in this report is that mixed-species biofilms comprised of three very different CF-associated pathogens (a Gram-positive species (SA), a Gram-negative species (PA), and a fungal species (CA)) display distinctly different compositional dynamics compared with their planktonic counterparts. In particular, we note that the species profiles in polymicrobial biofilms are consistent with both antagonistic and synergistic interactions. For example, irrespective of the substrate, adhered CA titres were lower in the polymicrobial biofilms compared with CA titres in biofilms from axenic CA cultures.

The primary limitation of *in vitro* models of infection is a lack of spatial organisation and exclusion of host cells present when utilising *in vivo* infection models. We therefore attempted to redress this issue through the introduction of EVPL tissue sections as biofilm substrata. Porcine airways share a remarkable degree of structural homology with human airways ( Judge *et al*., 2014; Rogers *et al*., 2008), and pig lungs are readily available from most butchers as a by-product of the food industry. Hence, the inclusion of *ex vivo* bronchiole tissue sections provide an ethically sustainable approach to introduce an element of host-microbe interaction and spatial organisation to the model ( Harrington *et al*., 2020; Sweeney *et al*., 2021). Indeed, we note that growth on different solid substrata had a profound effect on the compositional dynamics of polymicrobial biofilms, especially with regards to *S. aureus.* SA is an effective early coloniser of the CF airways ( Bogaert *et al*., 2004). When grown on EVPL sections, SA rapidly established a mono-species biofilm with significantly higher titres compared with the biofilm formed on agar chunks. This increased microbial attachment is consistent with the notion that SA adheres tightly to epithelial cells in the CF microenvironment ( Josse *et al*., 2017), but is less effective at adhering to abiotic surfaces (agar chunks). However, and whereas SA titres on the agar chunk substrata were stable between the 24 h and 96 h sampling points, the SA titres on EVPL sections displayed a substantial decline over this period. By contrast, PA titres in the same biofilms increased, suggesting that the PA progressively displaces SA on the tissue surface. This reciprocal response (in terms of cell titres) by PA and SA was not observed when agar chunks were used as a substratum, suggesting that recognition of both airway epithelial cells and SA are required to drive the increased competitiveness by PA.

Despite the introduction of some level of spatial organisation (in the form of EVPL tissue), the major physiological limitation of our model compared with *in vivo* systems is a lack of live host cells and the absence of any accompanying host-microbe interactions that may modulate microbial behaviour. Nevertheless, and through our comparison of EVPL tissue and agar chunks as biofilm substrata, we note two things. First, the diminution in SA titres in polymicrobial biofilms after 96 h growth on EVPL tissue – a diminution not seen on agar chunk substrata – suggests that microbial interaction with the host tissue may up-regulate the localised production of virulence factors and enhance interspecies competition ( Döring *et al*., 2011; Malhotra *et al*., 2019). The implication of this is that the growth of EVPL-associated biofilms in our continuous-flow model may enable the capture specific temporally-sensitive interactions between the host tissue and infecting microbes. Second, agar is a more suitable substratum for examining the long-term, steady-state growth of polymicrobial biofilms.

The ability to maintain very stable (in terms of composition) long-term polymicrobial biofilms in our setup using agar chunks as a solid substratum can be exploited for several other applications. First and most obviously, the setup can be used to investigate the species specificity (for example) of novel antimicrobial or anti-biofilm agents. Using our system, it is possible to test both a sustained treatment regimen (achieved by adding the test compound(s) at the desired concentration to the media reservoir) or a short-term treatment regimen, *via* adding a compound directly to the culture vessel through the injection ports. The latter may loosely mimic the metabolism and excretion of antimicrobials *in situ.* This degree of temporal control is not possible using existing *in vivo* or *in vitro* models. Second, new species or strain variants can be introduced to pre-established polymicrobial biofilms (e.g. addition of PA to a PA-negative community), allowing facile examination of the impact made by new species or variants. This is directly analogous to the situation seen in CF, where the acquisition of new microbial species or variants can lead to major prognostic changes in the patient. It is important to note that whenever a new species is introduced, the medium flowrate ( *Q*) may need to be re-optimized. If *Q* is too high, slower-growing organisms may be “washed out” and lost from the culture vessel. Conversely, if *Q* is too low, species will grow faster than the rate of media displacement, causing elements of the population to exhaust key nutrients and enter the stationary phase of growth.

## Conclusions

In summary, we present here a simple system for the study of CF-associated polymicrobial biofilms. Importantly, we have shown that polymicrobial biofilms display distinctly different population dynamics compared with the surrounding planktonic cells. Moreover, the compositional dynamics of the biofilms depends very much on the substratum employed. To further extend the utility of our model, we are currently adapting it for use with a wider range of CF-associated pathogens and testing the possibility of inoculating the system directly with CF patient-derived sputum.

## Data availability

### Underlying data

Figshare: Underlying data for ‘An *in vitro* model for the cultivation of polymicrobial biofilms under continuous-flow conditions’. https://doi.org/10.6084/m9.figshare.14974479.v1 ( O'Brien *et al*., 2021).

This project contains the following underlying data:

• EVPL Biofilms CFUs
• EVPL Supernatant CFUs
• Agar chunks Biofilm CFUs
• Agar chunks Supernatant CFUs
• Selective vs non-selective agar

Data are available under the terms of the Creative Commons Attribution 4.0 International license (CC-BY 4.0).
