# Supplementary material for: An in vitro model for the cultivation of polymicrobial biofilms under continuous-flow conditions
Source: F1000Res. 2021 Aug 13;10:801. [Version 1] doi: 10.12688/f1000research.55140.1 (PMC8442117; doi:10.12688/f1000research.55140.1)
Supplement: Peer review history

# Peer review: Reviewer response for version 1

**DOI:** 10.5256/f1000research.58690.r91903

**Published:** 2021-09-14

**Authors:** Lindsay R. Kalan

**Affiliations:** 1 Department of Medical Microbiology and Immunology, School of Medicine and Public Health, University of Wisconsin-Madison, Madison, WI, USA

The manuscript by O'brien *et al.* describes the development of an *in vitro* model for the study of polymicrobial biofilm, specifically focused on the CF lung environment. This work is timely as the importance of polymicrobial colonization, biofilm formation, and infection is increasingly recognized as important towards disease outcome. Here, the authors use artificial sputum in a continuous flow culture vessel to cultivate biofilms. They examined biofilm formation using two different substrates, agar or *ex vivo* porcine lung tissues. This allows the user to evaluate both biofilm growth on the substrate and measure planktonic cell populations in the surrounding media.

 The manuscript is well written, and the photographs of the apparatus are helpful to the reader who wishes to set this up in their own laboratory. The protocol provided is detailed and thorough although the production of ASM media seems cumbersome with some steps requiring first hand know-how that may introduce variability. For example, the filtration step is stated to take up to 2-3 days with the membrane being rinsed periodically, it is unclear how this might introduce contamination or impact the final media composition.

 I have no major comments, minor comments follow:

- The protocol states that each species in inoculated at an OD600 of 0.05. This could vary in the final cfu/mL depending on the species, especially fungi. It may be more informative to state the desired final inoculum in cfu/mL or total CFUs.
- The substrates are washed three times after incubation and before final quantification of biofilm. I wonder if these washes are ever quantified. This may be important to determine if the wash step disrupts significant amounts of biofilm for organisms that are loosely adherent or reaching maturity and may dislodge due to sheer pressure. This could possibly be the case with *S. aureus*.
- Figure 4 is not overly informative showing CFUs of overnight cultures. However, if the main point is that the selective media does not impact growth and quantification, the legend should state the selective media used for each species including any antibiotics/antifungals in the media.
- The authors state in the discussion that the model is amenable to longitudinal studies of polymicrobial biofilm for extended periods of time. However, this study only went out to 96 hrs. It is not clear how long or how stable these communities may be over longer periods. If the model allows for study over several days and even weeks this is very powerful. This information must be determined for each community, however the authors could address their experience with the three-member model community reported in this manuscript in the discussion.

**Are a suitable application and appropriate end-users identified?**

 Yes

**If applicable, is the statistical analysis and its interpretation appropriate?**

 Yes

**Are the 3Rs implications of the work described accurately?**

 Yes

Is the rationale for developing the new method (or application) clearly explained?

Yes

Is the description of the method technically sound?

Yes

Are the conclusions about the method and its performance adequately supported by the findings presented in the article?

Yes

If any results are presented, are all the source data underlying the results available to ensure full reproducibility?

Yes

Are sufficient details provided to allow replication of the method development and its use by others?

Yes

Reviewer Expertise:

Polymicrobial interactions, biofilm, chronic infection

I confirm that I have read this submission and believe that I have an appropriate level of expertise to confirm that it is of an acceptable scientific standard.

# Peer review: Reviewer response for version 1

**DOI:** 10.5256/f1000research.58690.r91897

**Published:** 2021-08-20

**Authors:** Daniel R. Neill

**Affiliations:** 1 Institute of Infection, Veterinary and Ecological Science, University of Liverpool, Liverpool, UK

O’Brien and colleagues describe a novel model system for the study of polymicrobial biofilms, under conditions designed to reflect those of the cystic fibrosis airways. The authors have built on their previous study that described a continuous-flow model for cultivation of mixed microbial populations. The neat addition, described here, is a metal gauze cage that can be loaded with substrata on which microbial biofilm communities can form. In the examples presented, cubes of agar or *ex vivo* porcine bronchioles are used. Culture in artificial sputum media provides environmental conditions reflective of the CF airways.

 This is a very well presented manuscript, with a carefully considered introduction and sufficient methodological detail to enable other users to establish the model in their own labs. The discussion describes further applications for the model that all appear realistic. I agree with the authors’ assertion that this model has potential to reduce or replace animal use for study of polymicrobial biofilms in CF and related conditions, albeit dependent upon the experimental question under consideration.

 This is primarily a methods paper, so the experimental findings are understandably limited. Nonetheless, I thought the observation of steady planktonic microbial density but fluctuating biofilm densities/composition was an interesting one. There is clear potential for further informative science to be undertaken using this model system.

 I have no major criticisms of the work. I thought the study had been well planned, carefully executed and nicely presented. I have no doubt that others with interests in polymicrobial infections of the CF lung will find the model useful, and I can see it being adapted for study of infection in other chronic lung conditions, as the authors suggest.

 Below, I include a few minor comments that the authors may wish to consider:

- The introduction mentions the limits of *in vitro *systems as being the lack of host cells and an immune system. I would add that most fail to capture the spatial heterogeneity of the respiratory tract. Chemical, nutritional, and microbial conditions differ between anatomical sites within the upper and lower airways and between different foci of infection within lungs. These differences may influence the dynamics of infection, the balance of competing pathogens in the airway space and may also contribute to treatment failure.
- The introduction states that “ *it should be noted that impaired immune clearance of microbes is an inherent feature of CF, which somewhat mitigates the lack of a functional immune system in in vitro models*.” I take the authors point on this, but it is an oversimplification. Inefficient immune clearance is not the same as having no immune response at all. There is still potential for interactions between soluble and cellular immune defences and bacterial pathogens to influence microbial phenotypes. The authors do partially address this point in their discussion, but I think this section of the intro is slightly misleading.
- For the presentation of results, I wondered whether CFU/cm ^2^ of substratum might be more informative than per ml? If this is too challenging to quantify, then per gram of agar/EVPL would at least account for differences in preparation between individual cubes.
- In the section on the microbial densities within biofilm, I was interested in whether there was much difference between biological replicates (i.e. between cubes). Does PA outcompete SA every time or does it depend on which gets a foothold first? Do you ever find cubes that are completely dominated by a single pathogen? This information is available within the source data file, but would be easier to digest in a graph presenting the biological replicates for each species side by side.
- The authors postulated explanations for the relative success of PA vs SA on EVPL are intriguing, but this section should acknowledge that only one strain of each species has been tested. PAO1 is fast growing compared to many PA isolates from CF and this may contribute to the observed outcomes.
- Although clearly beyond the scope of this manuscript, I was interested as to how the authors think the model may need to be adapted to better reflect the environmental conditions of the CF lung now that patients are moving over to CFTR modulator therapies?

**Are a suitable application and appropriate end-users identified?**

 Yes

**If applicable, is the statistical analysis and its interpretation appropriate?**

 Yes

**Are the 3Rs implications of the work described accurately?**

 Yes

Is the rationale for developing the new method (or application) clearly explained?

Yes

Is the description of the method technically sound?

Yes

Are the conclusions about the method and its performance adequately supported by the findings presented in the article?

Yes

If any results are presented, are all the source data underlying the results available to ensure full reproducibility?

Yes

Are sufficient details provided to allow replication of the method development and its use by others?

Yes

Reviewer Expertise:

Referee suggested by the NC3Rs for their scientific expertise and experience in assessing 3Rs impact. My research is focussed on bacterial infections of the respiratory tract, including those of the CF lung. I also have interests in development of in vitro and in vivo infection models that reflect respiratory tract environments.

I confirm that I have read this submission and believe that I have an appropriate level of expertise to confirm that it is of an acceptable scientific standard.
